# PI3K/AKT通路在非小细胞肺癌顺铂耐药中的作用

**DOI:** 10.3779/j.issn.1009-3419.2014.08.09

**Published:** 2014-08-20

**Authors:** 雨 张, 红玲 陆, 刚 徐

**Affiliations:** 1 563099 遵义，遵义医学院附属医院心胸外科 Departmen of Cardiothoracic Surgery, Affiliated Hospital of Zunyi Medical College, Zunyi 563099, China; 2 563099 遵义，遵义医学院生物化学教研室 Department of Biochemistry, Zunyi Medical College, Zunyi 563099, China

**Keywords:** 肺肿瘤, PI3K/Akt通路, 顺铂, 耐药, 分子机制, Lung neoplasms, PI3k/AKT pathway, Cisplatin, Resistance, Molecular mechanisms

## Abstract

研究表明, PI3K/AKT信号通路在非小细胞肺癌存在异常激活, 对肿瘤细胞的增殖、凋亡、存活、耐药等起着重要的作用。顺铂是临床最常用一线化疗药物, 但随着治疗的进展肿瘤对其耐药的现象越来越普遍, 严重制约其临床效果。顺铂耐药是多种机制共同参与的复杂过程, 其中PI3K/AKT通路或其组分持续被激活是非常重要的因素之一。本文对二者关系研究领域的进展作一综述。

## Abstract (en)

Accumulating evidences indicate that aberrant activation of PI3K/AKT pathway in non-small cell lung cancer plays a vital role in tumor cell proliferation, apoptosis, and survival including drug resistance.Cisplatin as first-line chemotherapy are in widespread clinical use in patients with non-small cell lung cancer, however, the development of cisplatin resistance significantly impedes its clinic efficacy.Cisplatin resistance is a complicated process that various mechanisms participating in to interact, of which PI3K/AKT pathway keeping sustained activated is one of the most important reasons.This article reviewed the progress of research on the relationship between PI3K/AKT pathway and cisplatin resistance.

肺癌是当前世界对人类健康和生命威胁最严重的肿瘤之一, 其发病率和死亡率已跃居各类恶性肿瘤的前列^[1]^。肺癌病例中, 80%-85%为非小细胞肺癌(non-small cell lung cancer, NSCLC), 尽管近年来癌症的分子靶向治疗取得了很大程度突破, 但目前以DNA毒性为主要细胞毒性的药物治疗手段仍然占据主导地位^[2]^。临床上根据患者肿瘤组织病理分型而选择相应药物治疗, 最理想的方案仍是以顺铂为主联合其他药物的治疗。然而即便如此, 联合用药对于Ⅳ期NSCLC的有效率不到30%, 3年生存率低于5%^[3]^。说明随着晚期肺癌患者化疗的逐渐开展, 产生了肿瘤细胞的耐药, 制约了抗肿瘤药物疗效的发挥, 影响化疗的预后^[4]^。据美国癌症协会估计, 90%以上因肿瘤死亡的患者在不同程度上受到耐药影响。顺铂的耐药是多因素、多通路作用的结果, 大致可总结为:①细胞内顺铂浓度降低; ②药物解毒作用增强; ③DNA修复机制改变; ④细胞凋亡通路改变^[3]^。这些机制中都涉及多种复杂的细胞信号转导通路, 其中PI3K/AKT/MTOR通路在人类肿瘤发生中出现异常调控现象^[5]^。该通路某些成分突变可导致通路功能改变, 不仅可以调节肿瘤细胞的增殖和存活, 还与肿瘤的侵袭转移及耐药密切相关^[6]^。因此, PI3K-Akt通路成为近年来研究的热点, 并被认为是化疗耐药治疗的新靶点。

## 1 PI3K/AKT/mTOR信号转导通路的组成及激活

磷脂酰肌醇3激酶(phosphatidylinositol 3-kinase, PI3K)是脂质激酶家族的成员, 是PI3K/Akt/mTOR通路中的始动因子。根据结构特点与底物特异性分为Ⅰ型、Ⅱ型、Ⅲ型, 其中Ⅰ型又分为ⅠA和ⅠB型。PI3K ⅠA可被RTK、G蛋白偶联受体(G protein-coupled receptors, GPCR)及小G蛋白如RAS等癌基因所激活, 而PI3K ⅠB则被GPCR特异性调控。PI3K ⅠA是由调控亚基P85和催化亚基P110组成的异二聚体。在哺乳动物中, *PIK3R1*、*PIK3R2*及*PIK3R3*基因分别编码p85α、p85β及p55γ, 统称P85。在生长因子刺激或RTK激活作用下, 导致PI3K被募集到细胞膜。P85通过其SH2区域在细胞质中与RTK磷酸化的酪氨酸残基结合, 释放出P110。PIK3CA、PIK3CB、PIK3CD则编码催化亚基P110的三种亚型p110α、p110β和p110δ, 活化的P110催化磷脂酰肌醇4, 5二磷酸转变为3, 4, 5-三磷酸磷脂酰肌醇(phosphatidylinositol 3, 4, 5-triphosphate, PIP3)。PTEN可以通过对PIP3去磷酸化还原其为PIP2而对PI3K/AKT通路起到明显抑制作用, 此为该通路重要的负调控基因。

AKT又称为蛋白激酶B(protein kinase B, PKB), 是PI3K下游的靶蛋白。AKT被PIP3募集而转位到细胞膜并与其PH区域结合, 导致构象改变暴露出丝氨酸、苏氨酸残基。3-磷酸肌醇依赖性蛋白激酶1(3-phosphoinositide-dependent protein kinase-1, PDK1)介导苏氨酸308位点磷酸化, 雷帕霉素靶蛋白复合物2(mTORC2)负责丝氨酸407位点磷酸化。AKT被激活后, 即可激活下游靶蛋白, 如糖原合酶激酶-3(glycogen synthase kinase 3, GSK3)、叉头转录因子(Forkhead transcription factor, FOXOs)、Bcl-2家族促凋亡成员(Bcl-2 family members associated death promoter, BAD)等, 发挥促细胞生长、抗凋亡作用^[7]^。

mTOR是PI3K/Akt通路的下游分子, 存在两种明显不同的复合物:mTORC1和mTORC2。mTORC1由mTORC催化亚基, Raptor、PRAS40与mLST8/GbL组成。mTORC2由mTOR、Rictor、mSIN1与mLST8/GbL组成。TSC1蛋白与TSC2蛋白形成复合物抑制Rheb从而抑制了mTORC1的活化。AKT介导磷酸化TSC2从而解除了复合物对Rheb的抑制, Rheb与GTP结合积累到一定程度完成mTORC1的激活。mTOR激活后可通过调节下游在翻译过程中有重要作用的两种蛋白, 真核细胞起始因子4E结合蛋白(eukaryotic initiation factor 4E-binding protein, 4E-BP1)与核糖体S6激酶1(ribosome protein subunit 6 kinase 1, S6K1), 从而起到监测养分供应、细胞能量水平、氧含量、有丝分裂信号等而达到调控细胞生长与增殖的作用^[8]^。

## 2 PI3K-Akt通路和肺癌顺铂耐药的关系

PI3K/AKT/mTOR通路的过度激活在以铂类药物为主的肿瘤耐药发展过程中起到至关重要的作用^[9, 10]^。而且根据此通路中的基因变异能够判断中国的晚期NSCLC患者采用铂类联合用药的效果是否理想^[11]^。

### 2.1 通路本身异常/持续激活状态

由于肺癌中多种基因突变可能通过作用于PI3K通路中某一组分而过度激活PI3K通路致使该通路在肺癌中多呈现异常调控现象^[12]^。

表皮生长因子受体(epidermal growth factor receptor, EGFR)、间变性淋巴瘤激酶(anaplastic lymphoma kinase, ALK)、鼠类肉瘤滤过性毒菌致癌同源体B1(vraf murine sarcoma viral oncogene homolog B1, BRAF)、鼠类肉瘤病毒癌基因(Kirsten rat sarcoma viral oncogene homolog, KRAS)、人类表皮生长因子受体2(human epidermalgrowth factor receptor-2, HER2)、磷脂酰肌醇3激酶催化亚单位α(phosphatidylinositol-3 kinase catalytic alpha, PIK3CA)、蛋白激酶B1(protein kinase B1, PKB1)、人丝裂原激活蛋白激酶激酶1(mitogen activated protein Kinase Kinase 1, MAP2K1)、原癌基因1酪氨酸激酶(oncogene 1 receptor tyrosine kinase, ROS1)这些基因已被证实是最为普遍存在于NSCLC中的突变基因^[13]^。其中*EGFR*、*KRAS*、*HER2*、*PIK3CA*、*AKT1*、*RAS*等基因突变与PI3K/AKT通路的异常活化状态有密切关系。PI3K Ⅰ型的激活相比于其他亚型对够维持肿瘤细胞的增殖与生存起到更重要的作用, 有研究发现PIK3CA过表达于NSCLC的原发肿瘤与其转移灶中^[14]^且常呈现扩增(12%-20%)以及突变状态(2%-5%), 伴随提高AKT的活性^[15]^。但是近来越来越多的证据支持其他基因也参与肿瘤发展^[16]^。PIK3R1亦常在NSCLC, 卵巢肿瘤以及结肠肿瘤中突变, 而且其低表达长预示该基因对肿瘤生长有抑制作用。

许多肿瘤中*PTEN*、*PIK3CA*、*PIK3R1*与*AKT*突变, 及PIK3CA与AKT1扩增都能过度激活AKT。激活状态的AKT(p-AKT)表达升高被证实存在于43%-90%的NSCLC与约50%的SCLC病例中, 在这其中存在1% AKT1突变^[17]^。p-AKT表达于早期的原发性肿瘤中的NSCLC患者提示预后差^[18]^。在80株NSCLC细胞中的13株细胞存在AKT的激活, 并且这13株中的12株细胞分别带有*EGFR*、*HER2*突变, *PIK3CA*扩增, *PTEN*丢失等异常^[19]^。p-AKT发挥其作用能够抑制促凋亡Bcl2家族BAD和BAX, 磷酸化Mdm2借由对抗P53介导的凋亡, 并且负调控Forkhead转录因子导致促细胞程序死亡蛋白减少^[17]^; 激活核转录因子kappa B(nuclear transcription factor kappa B, NF-κB), 启动抗凋亡基因Bcl-2、Bcl-XL等表达^[20]^; AKT直接磷酸化cAMP应答元件结合蛋白(cAMP response element bound protein, CREB), 诱导*Bcl-2*、*Bcl-XL*等相关基因表达等, 对抗化疗药物诱导肿瘤细胞凋亡作用而导致耐药的产生。已有研究^[21]^发现对顺铂耐药的肺癌细胞中*AKT1*基因扩增及表达过量是导致该肺癌细胞对于顺铂耐药的主要原因。

mTOR作为该通路一重要节点也被证实在肺癌细胞株中存在高表达状态^[22]^, 而且有研究表明其激活状态(p-mTOR)表达于90%的腺癌、60%的大细胞癌及40%的鳞状细胞癌中^[17]^。并且mTOR在早期NSCLC中表达提示预后差^[23]^。并且有报道^[24]^称mTORC2可能通过EGFR/mTORC2/NF-κB途径参与了恶性胶质瘤的耐药, 并且在肺癌耐药的研究中发现它与肿瘤耐药关系密切^[25]^。

PTEN作为该通路的重要的负调控因子, 常在众多肿瘤中由于突变、缺失或者转录后沉默等机制表达丢失^[26]^。在NSCLC中PTEN的完全性或部分表达丢失最为常见, 多是因为启动子甲基化导致转录后的沉默。由于PTEN作用减弱而导致PI3K/AKT通路功能上调, 从而参与多种机制导致的耐药发展。

### 2.2 靶向作用于PI3K/AKT通路各组分致耐药因素

#### 2.2.1 靶向作用酪氨酸蛋白激酶受体(receptor tyrosine kinase, RTK)

##### 2.2.1.1 IGFBP-3

胰岛素样生长因子结合蛋白3(insulin-like growth factor binding protein 3, IGFBP-3)能够结合IGF-1抑制其与一些普遍过表达与NSCLC中的RTK结合, 如IGF1R和EGFR^[27]^, 而抑制PI3K/AKT通路的激活。IGFBP-3多因启动子甲基化而在一些肿瘤中呈低表达, 这其中包括NSCLC。一项包含57例NSCLC患者及17例良性肺部病变患者的病理标本及血清研究发现, IGF1在肺癌患者病理组织标本以及血清中相比于良性病变患者明显升高, 而IGFBP-3表达则明显降低^[28]^。有报道^[29]^指出由于启动子甲基化致使IGFBP-3在NSCLC中的表达减少能够降低对顺铂的敏感性。而后进一步的研究证实, 在NSCLC细胞中, IGFBP-3由于启动子甲基化而导致表达下调或者丢失能够通过激活IGFIR而激活PI3K/AKT通路而导致顺铂耐药产生, 而且在胰腺癌细胞中也有相应的机制存在, 说明IGFBP-3对于IGFIR/AKT的调控影响顺铂敏感性机制可能普遍存在于人类肿瘤中^[30]^, 并且具有重要临床意义。

##### 2.2.1.2 Klotho

Klotho是一种新被发现的抗衰老基因, 但越来越多的被看做抑癌基因, 在肺癌与乳腺癌中能够抑制胰岛素样生长因子1(insulin-like growth factor 1, IGF-1)通路作用^[31, 32]^。但是在多种肿瘤细胞或者病理标本中多因启动子甲基化而被沉默表达, 并且恢复其活性后表现出明显对肿瘤生长的抑制作用^[33, 34]^。已经有对于手术切除的小细胞肺癌及大细胞肺癌肿瘤标本的研究^[35, 36]^显示, Klotho的表达预示了患者的预后良好。在NSCLC细胞中Klotho能够抑制IGF1磷酸化而抑制AKT的激活, 而肺癌细胞生长并诱导凋亡^[31]^, 在此基础上研究发现, 在顺铂耐药细胞株中Klotho的表达明显下调, 上调Klotho表达后发现, AKT活化导致的细胞对顺铂作用变得敏感; 敲除*Klotho*基因后, 耐药细胞的耐药性明显增强。这些结果揭示了Klotho能够通过影响PI3K通路的活性而使细胞对顺铂产生耐药。此外还发现, ERCC1在转染Klotho后的细胞中表达明显下降, 这亦说明Klotho能够通过影响ERCC1的表达而调控DNA修复的激活, 从而影响肿瘤细胞对于顺铂的耐药^[37]^。

##### 2.2.1.3 HtrA1

HtrA1是丝氨酸蛋白酶HtrA家族一员, 被越来越多的研究^[38]^证实在多种如黑色素瘤、卵巢、子宫内膜肿瘤中呈现明显下调表达, 而且HtrA1下调能够促进肿瘤发展, 尤其体现在肺癌中已经转移的淋巴结肿中, 说明HtrA1参与了肺癌发病过程^[39]^。多项研究显示HtrA1参与多种肿瘤细胞对化疗药物产生耐药作用的过程, 例如HtrA1调控紫杉醇与顺铂诱导的细胞毒性作用, HtrA1表达缺失能够导致卵巢肿瘤及胃癌细胞对化疗药物产生耐药^[40]^; 在一项关于人类胃癌肿瘤的研究^[41]^中发现中高水平表达HtrA1对顺铂的敏感性要优于低表达者。基于已有数据的考虑, Xu等的研究^[42]^中发现HtrA1在肺腺癌细胞中低表达能够激活PI3K/AKT通路而导致腺癌细胞呈现肿瘤干细胞样变而对顺铂产生耐药, 而其激活PI3K/AKT通路的方式可能类似于在卵巢癌细胞中的机制, 即HtrA1能够抑制EGFR活性从而抑制PI3K/AKT通路^[43]^, 当然还需要进一步验证。

#### 2.2.2 靶向作用AKT

##### 2.2.2.1 CABYR

睾丸癌(cancer testis, CT)抗原是一类在睾丸以及多种癌症中限制性表达的肿瘤抗原^[44]^。其过表达已有报道证实与多种肿瘤耐药相关^[45]^。

CABYR属于CT家族, 是一种钙结合酪氨酸磷酸化调节蛋白, 最先在人类精子中被提取。现已有报道CABYR蛋白在人类肺癌组织中异常增高, 但是在非癌组织中几乎不表达^[46]^。Qian等^[45]^的研究发现, 在高表达CABYR的NSCLC细胞株中沉默其表达后明显降低了p-AKT以及GSK-3β的表达。同时体内及体外研究发现, 下调CABYR-a/b不仅可增加肺癌细胞对化疗药物顺铂的敏感性, 而且亦能增强药物诱导细胞凋亡的效果。而转染激活的AKT进入细胞后耐药性恢复, 并且p-GSK-3β表达明显升高, 说明CABYR-a/b所介导肺癌细胞耐药是通过调控AKT通路实现的。

##### 2.2.2.2 ΔNp63α

p63是p53家族一员主要分为两类:一类为TAp63, 能够反式激活p53诱导细胞凋亡, 具有抑癌基因活性; 另一类为ΔNp63, 缺乏反式激活区域, 并且ΔNp63α是其中活性最强的亚组。ΔNp63α在肺癌、乳腺癌、子宫癌、前列腺癌中过表达提示预后差, 而且ΔNp63α主要在肺癌的鳞状细胞癌中以一种重要致癌基因作用过表达^[47]^。有报道称ΔNp63α高表达的患者往往对于顺铂为主的治疗容易产生耐受^[48]^, 并且在耐受顺铂的癌细胞中ΔNp63α的表达明显高于非耐受的癌细胞。在肺鳞状癌细胞中ΔNp63α的表达增多能够导致AKT1转录上调, 而且对顺铂产生耐药。最近发现ΔNp63α上调导致的对顺铂耐药可能是通过诱导miR-885-3p调控其下游重要功能基因包括AKT1而产生的^[49]^。

#### 2.2.3 靶向作用mTOR

##### 2.2.3.1 Twist

Twist是一种重要的EMT诱导因子, 能够下调表皮细胞标记物(E-cadherin), 上调间质细胞特征蛋白(fibronectin、N-cadherin、vimentin), 促进肿瘤的侵袭与转移。Twist1在大部分健康成人组织中不表达, 而在多种如黑色素瘤、子宫、前列腺、乳腺当然包括肺肿瘤组织中过表达^[50]^。有数据显示, 在一项包含87例NSCLC患者的肿瘤切除标本中, Twist1过表达率达36.8%, 而且其过表达预示复发间歇时间更短^[51]^。Hui等^[52]^关于68例肺腺癌和肺鳞癌标本及8例癌旁组织标本的研究发现, Twist的表达情况与肺癌的分化程度相关, 低分化的肺癌Twist高表达, 中高分化的肺癌其阳性表达。已有研究^[53]^发现, 利用siRNA对抗Twist表达能够增加A549细胞对于顺铂的敏感性, 进一步发现Twist1被敲除后, 肺癌细胞对顺铂敏感性增强; 抗凋亡Mcl-1蛋白明显下调; mTOR活性明显被抑制; 细胞中p21Waf1/CIP1上调。这些结果证明, Twist通过减少依赖mTOR/S6K1途径的Mcl-1表达, 增强顺铂对癌细胞的毒性作用。提示以mTOR与EMT交叉调控分子作为靶向目标可能成为有效的抗癌手段^[54]^。

##### 2.2.3.2 Redd1的过表达

Redd1也称为RTP801, 起初是被看作一种应激基因, 受低氧、DNA损伤、糖皮质激素治疗等刺激。Redd1在不同种类细胞中对各种刺激的应答体现出促生长或促凋亡双重作用。Redd1的表达能够使TSC2/14-3-3复合物解离从而激活TSC1/2, TSC1/2激活后发挥其抑制mTORC1的作用^[55]^。有研究^[56]^指出, Redd1过表达能够通过抑制mTOR而抑制NSCLC细胞的侵袭力。然而在Jin等的研究中, REDD1的表达抑制mTORC1的激活, 而且能够诱导AKT的完全磷酸化, 导致肿瘤细胞对顺铂耐药性增加。这些结果表明, AKT激活, mTORC1的抑制和持续Redd1的过度表达, 在细胞生存与对顺铂的耐受中起到重要作用。REDD1诱导的AKT磷酸化可能是通过能够诱导mTORC1抑制的负反馈抑制而出现的, 其作用机制尚有待进一步研究^[57]^。

### 2.3 PI3K/AKT通路下游耐药因素

#### 2.3.1 NF-κB

NF-κB是一种广泛存在的转录因子, 由同源或者异源P50与P65组成, 正常情况下与κB抑制蛋白(inhibitor of NF-κB, IκB)结合存在于细胞质中。众多生长因子受体可以通过PI3K/AKT通路激活NF-κB, p-AKT可直接磷酸化IκB致使其被泛素化而降解, NF-κB则被释放而转位至细胞核被激活, 启动其下游基因转录, 诱导血管生成, 细胞增殖等, 参与肿瘤的发生。NF-κB及其下游基因参与多种癌症发生^[58]^, 且其激活状态被发现存在于多种肿瘤细胞中包括肺癌细胞^[59]^。有研究^[60]^证实顺铂在作用A549细胞时能够产生表皮生长因子样作用而使得EGFR磷酸化而激活PI3K/AKT/NF-κB通路而上调NF-κB表达, 而后使得A549细胞对于顺铂产生耐药。这一研究结果提示顺铂可能诱导EGFR激活, 而通过影响NF-κB导致顺铂耐药。LI等通过对A549细胞模拟低氧环境后发现, NF-κB的激活后并未降低A549细胞生存率与顺铂IC50, 相反, 抑制低氧环境诱导的NF-κB激活可逆转低氧环境诱导的顺铂耐药^[61]^。

#### 2.3.2 *CA916798*基因

研究^[62]^发现, 通过抑制消减杂交(suppression subtractive hybridization, SSH)技术对比耐顺铂肺腺癌系与普通腺癌系, 发现一个在耐顺铂细胞系中明显高表达的基因并命名为CA916798。随后的研究^[63]^证实, 在对顺铂敏感的H446细胞中上调CA916798, 细胞可产生明显的耐药性; 在耐顺铂的A549细胞中下调该基因, 则其耐药性被逆转, 提示CA916798与顺铂耐药有密切关系。Qi等^[64]^为了阐清其耐药机制进一步研究发现, 阻断PI3K/AKT/mTOR通路后, CA916798的mRNA水平明显下降, 初步证实CA916798位于该通路下游, 受其调节转录。近期研究^[25]^证实了CA916798位于PI3K/AKT/mTOR下游, 而且是由AKT1依赖mTORC2途径调控的。

### 2.4 miRNA影响PI3K/AKT通路与耐药关系

MicroRNA是一类非编码蛋白的RNA, 能够与互补的靶信使RNA结合, 通过降解mRNA, 或抑制其翻译成蛋白而导致转录后蛋白表达的沉默^[65]^。在人类基因组中大约有800种-1, 000种miRNA, 虽然只占到人类基因组总数的很小一部分, 但它们在细胞生长、分化、凋亡、动力、恶变等过程中起到非常重要的作用^[66]^。

有研究^[67]^表明, MiR-21在很多人类恶性肿瘤包括NSCLC中过表达, 并且其过表达与TNM分期和淋巴结转移都有密切关系^[68]^。Liu等^[69]^发现, 抑制miRNA-21的表达能够诱导NSCLC细胞凋亡、转移与侵袭, 而且能够明显增加NSCLC细胞对顺铂的敏感性是通过转录后调控PTEN发挥的作用。同样有研究发现miR-92b水平与顺铂耐药同样相关, 而且miR-92b在NSCLC中也同样高表达, 敲除miR-92b后细胞生长被抑制, 而A549/DDP细胞对于顺铂的敏感性大大增加。进一步的研究^[70]^发现miR-92b靶向作用于PTEN, 导致PTEN的下调, 从而提示, miR-92b也可作为一种癌基因对细胞生长、耐药起到重要作用。

Bian等^[71]^的研究发现, NSCLC相比于正常肺组织中miRNA-451频繁下调, 猜测miRNA-451可能是一种抑癌基因, 而且miRNA-451上调能够抑制AKT的激活及倒置Bcl-2/Bax比例, 从而增强依赖caspase-3途径的细胞凋亡。此外, 无论在体内还是体外实验, 上调miRNA-451都能明显增加癌细胞对于顺铂的敏感性。Qiu等发现, miR-503在对顺铂耐药的NSCLC细胞中表达减少, 而过表达miR-503能够增强A549/DDP细胞对于顺铂的敏感性, 进一步的研究发现, miR-503能够特异性作用于PI3K通路下游重要的抗凋亡基因*Bcl-2*, 说明miR-503在一定程度上通过靶向作用于Bcl-2调节细胞生长与对顺铂的耐药^[72]^。另外亦有报道称PI3K p85亚基是miR-503直接作用靶点^[73]^。另有报道发现, miR-200bc/429复合物^[74]^与miR-181b^[75]^都是靶向作用于BCL-2而影响肺癌细胞对顺铂的耐药。

## 3 结语

目前国内外关注较多的是抗肿瘤作用的新靶点和新型抗肿瘤剂或手段, 包括以细胞信号转导分子为靶点的信号转导通路抑制剂。PI3K/AKT通路在NSCLC细胞中常呈激活状态而过度发挥其作用^[76]^, 与NSCLC细胞对化疗药物产生耐药关系密切, 所以针对该通路靶点的抑制剂应运而生。分别有针对PI3K1A的抑制剂, 如XL147、PX­866、BKM120、GDC­0941、BAY806946、GSK2126458和CH5132799等; AKT抑制剂, 分为ATP竞争性抑制剂如AZD5363与变构性抑制剂如MK-2206等; mTOR抑制剂雷帕霉素及mTORC1/2抑制剂如OSI027;PI3K-mTOR双重抑制剂BEZ235、GDC­0980、SF1126等。理论上讲, 任何阻断PI3K/Akt通路或者其上下游的抑制剂都可以治疗NSCLC, 并且这种抑制剂毒副作用小, 治疗效果明显, 抑制PI3K/Akt通路还可以逆转由该通路活化引起的化疗耐药和不良预后。目前, 有些PI3K-Akt信号通路抑制剂已经应用于临床, 但效果尚不理想。因此, 仍需进行大量的临床和基础实验研究。深入了解PI3K/Akt信号通路在NSCLC耐药中的作用机制可以有助于开发新的分子靶向药物, 为NSCLC及其他耐药肿瘤治疗带来希望。
